# A non-pediocin low molecular weight antimicrobial peptide produced by *Pediococcus pentosaceus* strain IE-3 shows increased activity under reducing environment

**DOI:** 10.1186/s12866-014-0226-2

**Published:** 2014-08-27

**Authors:** Pradip K Singh, Shalley Sharma, Annu Kumari, Suresh Korpole

**Affiliations:** MTCC and Gene Bank, CSIR-Institute of Microbial Technology, Sector 39A, Chandigarh 160036, India

**Keywords:** *Pediococcus*, LMW antimicrobial peptide, MALDI-TOF MS, DTT effect

## Abstract

**Background:**

Species of the genus *Pediococcus* are known to produce antimicrobial peptides such as pediocin-like bacteriocins that contain YGNGVXC as a conserved motif at their N-terminus. Until now, the molecular weight of various bacteriocins produced by different strains of the genus *Pediococcus* have been found to vary between 2.7 to 4.6 kD. In the present study, we characterized an antimicrobial peptide produced by *P. pentosaceus* strain IE-3.

**Results:**

Antimicrobial peptide was isolated and purified from the supernatant of *P. pentosaceus* strain IE-3 grown for 48 h using cation exchange chromatography and reversed-phase high-performance liquid chromatography (RP-HPLC) techniques. While MALDI-TOF MS experiments determined the precise molecular mass of the peptide to be 1701.00 Da, the *de novo* sequence (APVPFSCTRGCLTHLV) of the peptide revealed no similarity with reported pediocins and did not contain the YGNGVXC conserved motif. Unlike pediocin-like bacteriocins, the low molecular weight peptide (LMW) showed resistance to different proteases. Moreover, peptide treated with reducing agent like dithiothreitol (DTT) exhibited increased activity against both Gram-positive and Gram-negative test strains in comparison to native peptide. However, peptide treated with oxidizing agent such as hydrogen peroxide (H_2_O_2_) did not show any antimicrobial activity.

**Conclusion:**

To our knowledge this is the lowest molecular weight peptide produced by members of the genus *Pediococcus*. The low molecular weight peptide shared amino acid arrangement with N-terminal sequence of Class IIa, pediocin-like bacteriocins and showed increased activity under reducing conditions. Antimicrobial peptides active under reduced conditions are valuable for the preservation of processed foods like meat, dairy and canned foods where low redox potential prevails.

## Background

Bacteria produces different kinds of antimicrobial substances including ribosomally synthesized bacteriocins and non-ribosomally synthesized antibiotics or lipopeptides as a part of their defense strategies in complex environments such as fermented foods and the human gut. Members belonging to the lactic acid bacteria (LAB) family with ability to produce bacteriocins are frequently found in these environments [1]. LAB strains are recognized as GRAS (Generally Regarded As Safe) microorganisms and have been studied in detail for biotechnological applications together with the bacteriocins produced by these strains [2,3]. Members of the genus *Pediococcus* are classified within the LAB family and are reported to produce bacteriocins without post-translational modifications that are classified under class II bacteriocins [4,5]. The bacteriocins classified under class IIa are called as pediocin-like bacteriocins because the first antimicrobial peptide of this class (pediocin PA-1) was isolated from *Pediococcus* sp. [6]. They include variable size peptides ranging from 2.7 to 4.6 kDa [7–9] with high sequence homology, disulfide bonds and a conserved motif YGNGVXC in their N-terminal domain [10]. However, bacteriocins lacking the consensus motif are also classified under pediocin-like bacteriocins [2]. Initially pediocin-like bacteriocins were reported to be produced by members of the genus *Pediococcus* [10] but later were also isolated from members of other genera like *Lactobacillus*, *Enterococcus* and *Bacillus* [11–14]. Since pediocin-like bacteriocins are well-known to inhibit the growth of food spoilage and pathogenic bacteria *Listeria monocytogenes*, they are also termed as anti-listerial bacteriocins and considered as potential antimicrobial additives for food preservation. Though pediocin producing members of the genus *Pediococcus* are largely isolated from dairy products, they have also been reported from diverse environments including human stool sample [15,16]. However, pediocin-like bacteriocins produced by different isolates exhibited 40-60% similarity in their amino acid sequence [10]. Among the known variants of pediocin-like bacteriocins, pediocin PA-1 is well-studied 4.6 kDa antimicrobial peptide with thermo-stability and wide pH range activity [17]. Nevertheless, it was inactivated by proteases like pepsin, trypsin, chymotrypsin, proteinase K and pronase E [10]. Further, structure of the pediocin PA-1 revealed presence of two β-strands connected by a β-hairpin made up of five amino acid residues in their N-terminal sequence that play an important role in antimicrobial activity [18–20]. In this study, we describe the isolation, purification and characterization of a novel antimicrobial peptide produced by *P. pentosaceus* strain IE-3 isolated from a dairy effluent sample [21].

## Results and discussion

### Growth conditions and antibacterial activity assay

**Figure 1:**
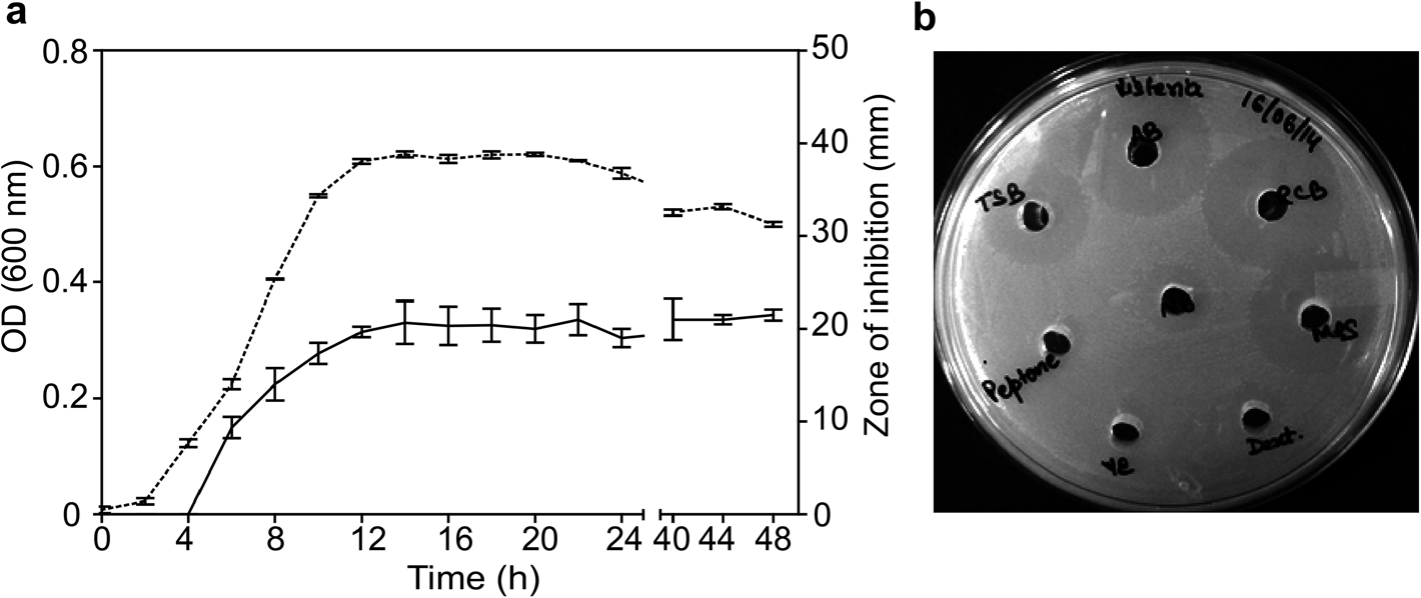
**Antimicrobial production by** ***P. pentosaceus*** **strain IE-3. (a)** Correlation between antimicrobial peptide production and growth of strain IE-3. Growth measured as OD at 600 nm (dotted lines), bacteriocin production as zone of inhibition (continuous line). Error bars shows ± SD for triplicate experiments. Culture was grown in anaerobic broth under anaerobic conditions at 30°C on a shaker incubator. **(b)** Antimicrobial assay of 24 h cell free fermented broth obtained by growing strain IE-3 on different media.

*P. pentosaceus* strain IE-3 showed very little growth while grown in aerobic conditions and optimal growth was observed under anaerobic conditions. The 48 h cell free fermented broth (CFB) of *P. pentosaceus* strain IE-3 grown in anaerobic broth displayed antimicrobial activity against different indicator strains in well diffusion assay (Table 1). In contrast to typical narrow spectrum activity shown by pediocin-like bacteriocins [10], the antimicrobial peptide produced by strain IE-3 inhibited growth of Gram-positive and Gram-negative indicator strains. The most sensitive strain among the test strains was *Micrococcus luteus* that showed a 26 mm zone of inhibition. There was no activity observed against other strains of *Pediococcus*, yeasts and fungi. A curve displaying antimicrobial production versus bacterial growth showed that the antimicrobial peptide production was initiated during early log phase (6 h of incubation) which increased to a maximum level by initial stationary phase (14 h) and remained constant thereafter (Figure 1a). Antimicrobial activity was obtained when the *P. pentosaceus* strain IE-3 was grown in different media including minimal medium with optimal production obtained in media like anaerobic broth, MRS and reinforced clostridial broth, the latter containing reducing agents (Figure 1b). Significant delay was observed to reach exponential growth phase by strain IE-3 while growing in minimal media that resulted in slow antimicrobial production (data not shown).

**Table 1:** **Antimicrobial activity of the cell free fermented broth (CFB) of 48 h grown culture against various test strains (mean values of triplicate experiments)**

**Test strain**	**Inhibition zone using CFB (mm)**
**Gram-positive**	
*Listeria monocytogenes* (MTCC 839)	13
*Lactobacillus plantarum* (MTCC 2621)	10
*Clostridium bifermentas* (MTCC 11273)	10
*Clostridium sordelli* (MTCC 11072)	12
*Bacillus subtilis* (MTCC 121)	<10
*Staphylococcus aureus* (MTCC 1430)	10
*Micrococcus luteus* (MTCC 106)	26
*Pediococcus acidilactici* (MTCC 7442)	-
*P. pentosaceus* (MTCC 9484)	-
*P. pentosaceus* (MTCC 10308)	-
**Gram-negative**	
*Vibrio cholera* (MTCC 3904)	15
*Escherichia coli* (MTCC 1610)	<10
*Pseudomonas aeruginosa* (MTCC 1934)	10
*Serratia marcescens* (MTCC 97)	-
**Fungi**	
*Candida albicans* (MTCC 183)	-
*Asperigillus flavus* (MTCC 8188)	-

-, no activity.

### Purification of antimicrobial peptide

**Figure 2:**
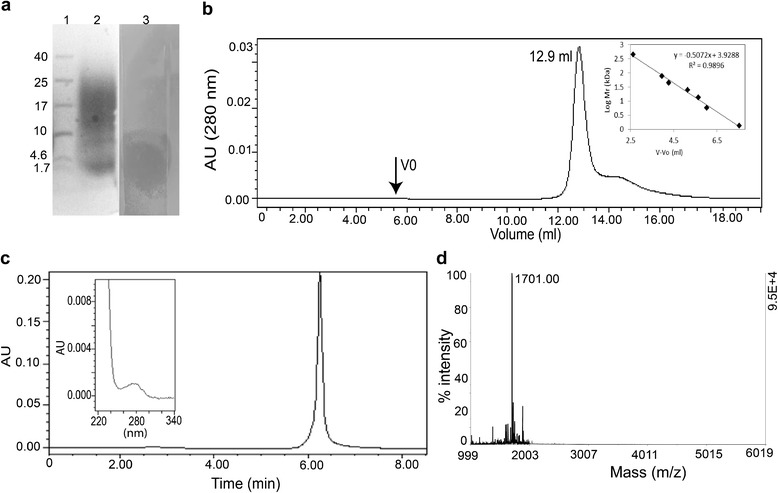
**Characterization of low molecular weight antimicrobial peptide produced by** ***P. pentosaceus*** **strain IE-3. (a)** In-gel activity assay of crude extract on 18% tricine SDS-PAGE gel, lane 1 contains low molecular weight protein marker, lane 2 crude extract obtained from Diaion HP20 and lane 3 showing antimicrobial activity against *L. monocytogenes* MTCC 839 **(b)** Size determination by gel filtration chromatography of cation exchange purified peptide along with standard graph (of known molecular weight proteins depicts low molecular size). **(c)** Reverse-phase HPLC profile of purified antimicrobial peptide and inset showing the absorbance between 260–280 nm. **(d)** Intact molecular mass showing as 1701.00 Da in MALDI-TOF analysis.

The crude extract obtained by Diaion HP20 chromatography showed significant increase in antimicrobial activity compared to CFB. Gel electrophoresis analysis of this extract displayed a peptide band between 1.7 and 4.5 kDa on tricine SDS-PAGE that showed antimicrobial activity against *L. monocytogenes* in in-gel activity assay (Figure 2a). Direct detection of antimicrobial activity by in-gel activity assay revealed that the inhibition was caused by a low molecular weight (LMW) peptide. The extract was purified on a cation exchange column and the active fraction obtained was used for gel filtration chromatography analysis that anticipated the molecular mass to be in the range of 2.0 - 5.5 kDa (Figure 2b). The purified peptide showed a single peak in reversed phase HPLC with absorbance between 260–280 nm (Figure 2c) that may be due to the presence of aromatic amino acids like phenylalanine. During storage of the purified peptide at room temperature significant reduction in antimicrobial activity was observed within 24 h, but was stable when stored at −20°C. Subsequently, it was found that the loss of antimicrobial activity was due to oxidation of peptide as observed for pediocin-like bacteriocins [22].

### Molecular mass analysis and *de novo* sequencing of LMW peptide

**Figure 3:**
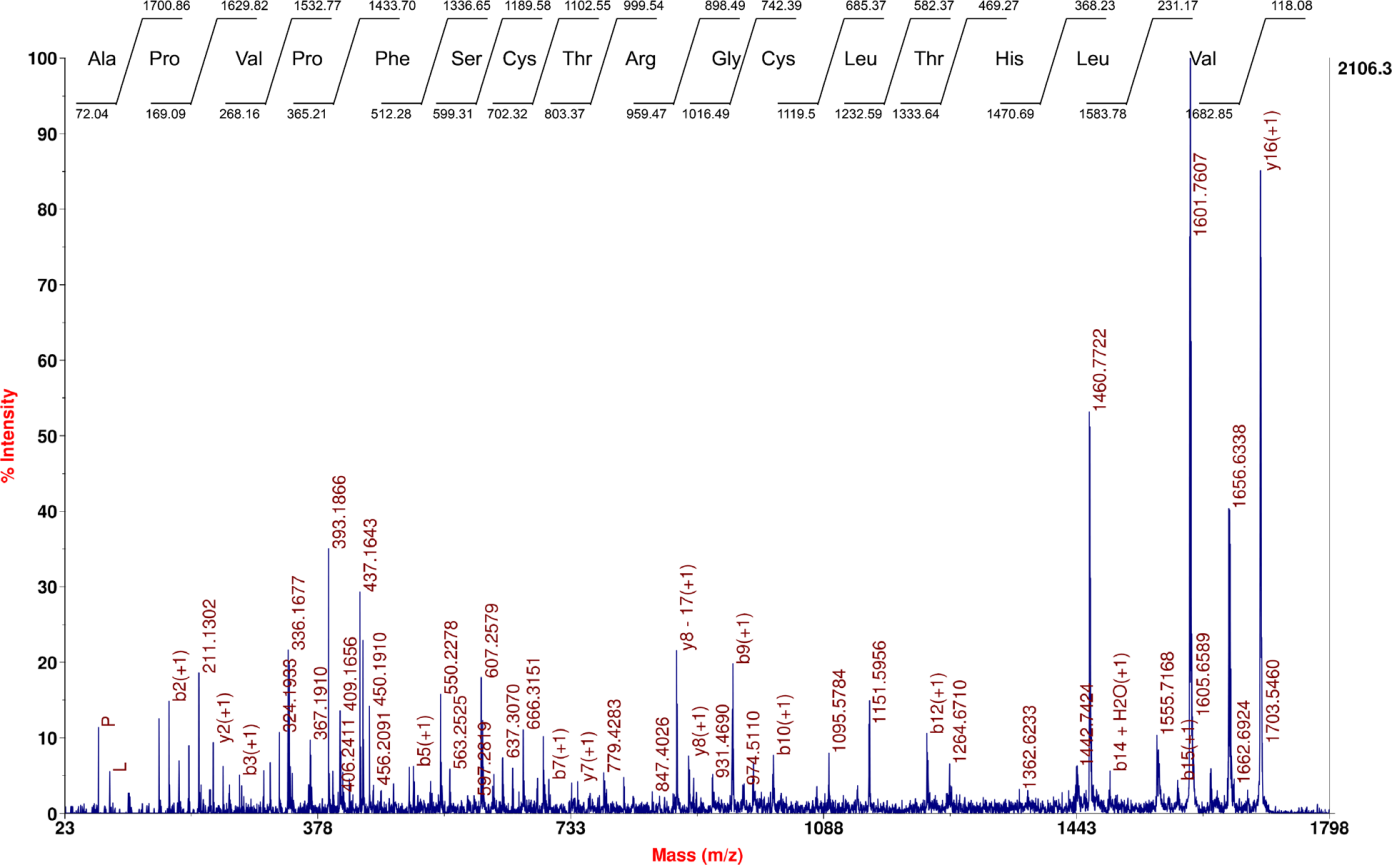
***De novo*** **sequence derived for the antimicrobial peptide generated by** ***de*** **-** ***novo*** **explorer of AB Sciex with highest score value (b ion values shown at bottom and y ion values at top).**

**Figure 4:**
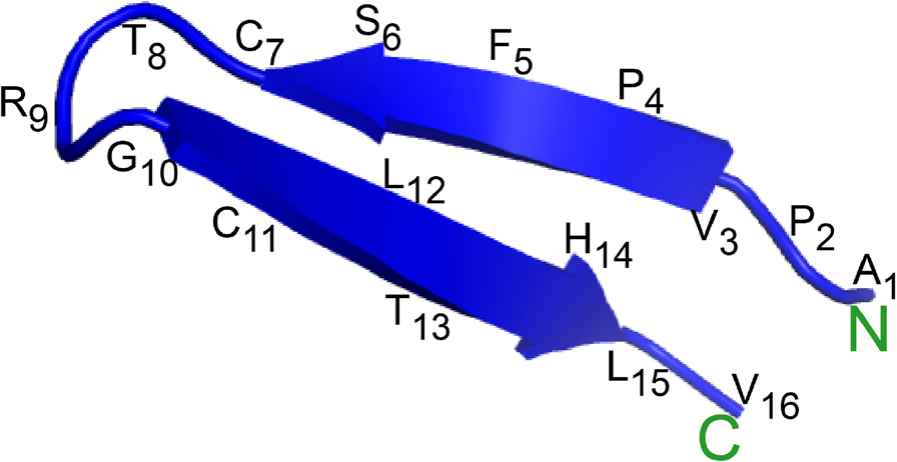
**Predicted 3-dimensional structure of** ***de novo*** **sequence obtained for low molecular weight antimicrobial peptide showing the presence of antiparallel β-sheets.**

The molecular mass for LMW antimicrobial peptide was determined as 1701.00 Da (Figure 2d) by MALDI-TOF MS. The primary structure of the peptide by MS/MS sequencing revealed the sequence as APVPFSCTRGCLTHLV with high score value of 47.59 (Figure 3). The mass obtained in MALDI-TOF is in agreement with the estimated theoretical average mass (1701.03 Da) obtained for the sequence. Minor differences in mass may be due to the instrument error which deviates up to 50 ppm. Further, bioinformatics analysis of the sequence did not show any significant similarity with known pediocin-like bacteriocins or other bacterial AMPs available with databases like Bactibase [23,24] or Collection of Antimicrobial Peptide (CAMP) database [25]. In fact, the *de novo* sequence was used for blast analysis against the published genome of strain IE-3, but could not find any significant blast hit covering the entire peptide sequence in the annotated proteins. Further, genome sequence analysis to find the ORF coding this peptide did not show any significant similarity. In this regard, it is pertinent to note that the genome of IE-3 is a draft version, hence, putative region encoding the small peptide may have missed in the draft genome due to gaps formed during the assembly of raw sequence data (as contig cutoff size of ≥500 nucleotides was used). Considering ambiguities in *de novo* sequencing, search was also made by replacing Leu residue with Ile as well as other *de novo* sequences obtained with low score values, however, no ORF was found in genome sequence. As no ORF detected in genome, it is anticipated that antimicrobial peptide might be produced from medium components by the strain IE-3. Nevertheless, synthesis of peptide from the medium components is ruled out as the peptide production was observed in minimal medium containing an inorganic nitrogen source. Though the *de novo* sequence similarity search using APD2 [26] revealed low similarity (37% similarity) with the eukaryotic antimicrobial peptide, temporin LTb, it did not show any conserved motifs observed for temporins [27]. Antimicrobial peptide prediction analysis [26] of the *de novo* sequence suggested that the peptide could be a potential antimicrobial peptide with the presence of cationic, aromatic and hydrophobic amino acids, along with two cysteine residues. Moreover, this LMW peptide shares an amino acid arrangement with N-terminal sequence of other pediocin-like bacteriocins such as pediocin PA-1 [9,28]. Remarkably, the five amino acids (CTRGC) of the peptide showed amino acids pattern similarity with β-hairpin composition (CTKSGC) of pediocin-like bacteriocins [29] where the positively charged amino acid play crucial role in antimicrobial activity [10]. In fact, addition of a positively charged amino acid within this patch showed significant increase in antimicrobial activity of pediocin PA-1 [30]. Additionally, structure prediction analysis for the LMW peptide showed antiparallel β-sheet confirmation (Figure 4) where hydrophobic and positively charged amino acids are enclosed by two cysteine residues (Cyt7-Cyt11).

### Effect of pH, temperature, proteolytic enzymes, reducing agent and H_2_O_2_ on antimicrobial activity

**Figure 5:**
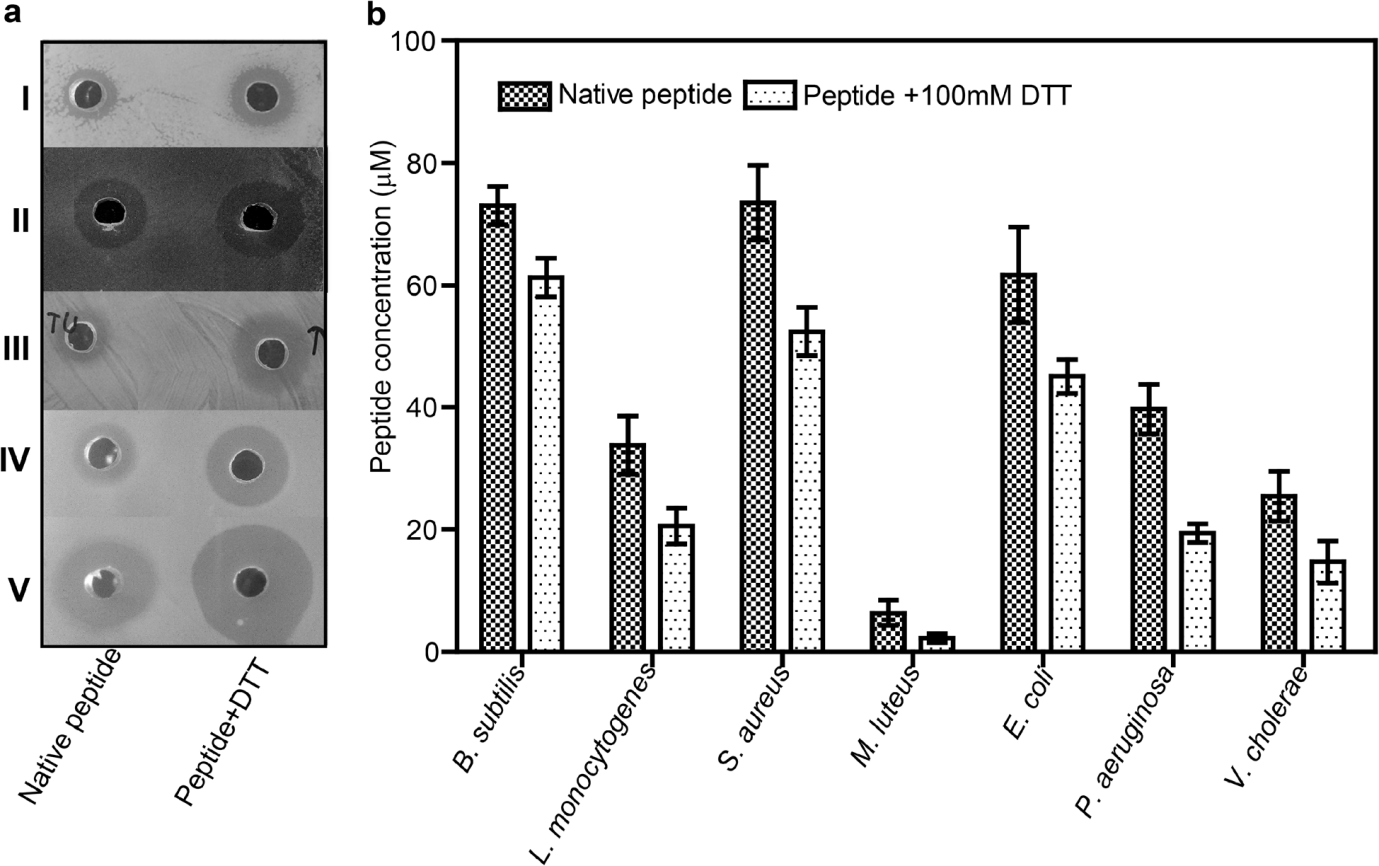
**Antimicrobial activity assay of native and DTT (100 mM) treated LMW peptides against Gram-positive and Gram-negative (I,** ***B. subtilis*** **; II,** ***L. monocytogenes*** **; III,** ***E. coli*** **; IV,** ***P. aeruginosa*** **and V,** ***V. cholera*** **) indicator strains (a).** Comparison of MIC values against various test strains **(b)**. Standard deviation (SD) is shown as error bars; significant difference between DTT treatment and control with p < 0.05 was observed in two independent experiments performed in triplicates.

The LMW antimicrobial peptide was found to be thermo-stable as there was no reduction observed in its antimicrobial activity even after 30 min of incubation at 100°C. However, it displayed sensitivity towards the pH as the maximum activity was observed at pH 5 and significant loss was found at pH 8 and above (Table 2). Unlike pediocin-like bacteriocins, the low molecular weight peptide in this study was found to be resistant to proteolytic cleavage as an antimicrobial assay performed upon incubation with proteolytic enzymes showed no reduction in activity. Stable activity upon treatment with amylase revealed the absence of any sugar moiety associated with inhibition activity of the bacteriocin. Interestingly, the peptide showed significant increase in antimicrobial activity when it was tested upon incubation with DTT (Figure 5a). The increase in activity was observed with the increase in DTT concentration up to 150 mM (Table 2). However, peptide incubated with H_2_O_2_ did not show any antimicrobial activity confirming the inactivation of LMW peptide upon oxidation. Results of control DTT experiments showed no effect on the growth of indicator strains.

**Table 2:** **Influence of different pH values and DTT concentrations on antimicrobial activity of the LMW peptide produced by** ***P. pentosaceus*** **strain IE-3**

**Treatments**	**Reaction condition**	**Residual activity (%)**
**pH**		
2	Overnight/ RT	100.0
3	Overnight/ RT	100.0
4	Overnight/ RT	100.0
5	Overnight/ RT	100.0
6	Overnight/ RT	87.5
7	Overnight/ RT	75.0
8	Overnight/ RT	37.5
9	Overnight/ RT	25.0
10	Overnight/ RT	12.5
**DTT concentration**		
0 mM	1 h/RT	100.0
50 mM	1 h/RT	125.0
100 mM	1 h/RT	143.7
150 mM	1 h/RT	143.7

RT, room temperature.

### Determination of minimum inhibitory concentration of the LMW peptide

Determination of minimum inhibitory concentration (MIC) for various indicator organisms revealed that the peptide was most active against *M. luteus* of Gram-positive strains with an MIC value of 6.3 μM. Among the Gram-negative strains, *V. cholera* growth was inhibited at 25.4 μM concentration. The MIC values observed for the peptide were higher when compared to other pediocin-like bacteriocins, however, MIC determined for peptide treated with DTT were found to be significantly lower than the native peptide (Figure 5b). Again, *M. luteus*, *L. monocytogenes* and *V. cholera* were observed as the most sensitive, however, test strains like *B. subtilis* and *E. coli* were inhibited more efficiently with the DTT treated peptide compared to native peptide. Hemolysis of rabbit RBCs was not observed at concentrations up to 100 μM of peptide.

## Conclusions

Although production of LMW antimicrobial peptides from different bacteria was reported in the literature, no peptide of less than 2.5 kDa was reported from *Pediococcus* species. Pediocin-like bacteriocins are produced by the pediocin biosynthetic gene cluster *pedABCD* that are highly conserved among *Pediococcus* strains, however, strains like *P. acidilactici* did not produce any antimicrobial substance though it contained pediocin biosynthetic gene cluster. Similarly, the draft genome sequence of strain IE-3 in this study showed presence of pediocin biosynthetic gene cluster *pedABCD* but did not produce any pediocin-like bacteriocin. We conclude that *P. pentosaceus* strain IE-3 produces a LMW antimicrobial peptide with broad spectrum antimicrobial activity that is resistant to proteases. Therefore, it may be used effectively against food spoilage bacteria and developed as an efficient preservative for processed foods in food industry.

## Methods

### Bacterial strains and growth media

The antimicrobial producing bacterial strain IE-3 was isolated from a dairy industry effluent sample. The draft genome sequence of strain IE-3 has been published earlier [21]. All test strains used in the present study were obtained from Microbial Type Culture Collection and Gene Bank (MTCC and Gene Bank), CSIR-Institute of Microbial Technology, Chandigarh, India. Indicator strains like, *Bacillus subtilis* MTCC 121, *Staphylococcus aureus* MTCC 1430, *Micrococcus luteus* MTCC 106 *Pseudomonas aeruginosa* MTCC 1934, and *Escherichia coli* MTCC 1610 were grown on nutrient agar (M001, Himedia, India), *Vibrio cholerae* MTCC 3904 was on LB medium (M1151, Himedia, India). Brain heart infusion agar (M1611, Himedia, India) was used to cultivate *Listeria monocytogenes* MTCC 839 and MRS medium (M641, Himedia, India) for *Lactobacillus plantarum* MTCC 2621. *Clostridium bifermentans* MTCC 11273, *C. sordelli* MTCC 11072, *Pediococcus acidilactici* MTCC 7442, *P. pentosaceus* MTCC 3817 and *P. pentosaceus* MTCC 9484 were grown on anaerobic agar (M228, Himedia, India). Among the eukaryotic test strains while *Candida albicans* MTCC 1637 was grown on YEPD medium (G038, Himedia, India), Czapek yeast extract agar (M1335, Himedia, India) was used to cultivate *Aspergillus flavus* MTCC8188. To test the influence of growth medium on antimicrobial production strain IE-3 was grown on nutrient broth (M002, Himedia, India), tryptone soya broth (LQ508, Himedia, India), reinforced clostridial broth (M443, Himedia, India), MRS broth (M369, Himedia, India) and minimal medium. Composition of anaerobic broth used for bacteriocin production contains (per liter) casein enzymic hydrolysate, 20.0 g; dextrose, 10.0 g; sodium chloride, 5.0 g; sodium thioglycollate, 2.0 g; sodium formaldehyde sulphoxylate 1.0 g; methylene blue, 0.002 g and pH adjusted to 7.2 ± 0.2. The minimal medium composed of (per liter) K_2_HPO_4_, 0.5 g; (NH_4_)_2_SO_4_, 0.5 g; MgSO_4_. 7H_2_O, 0.1 g; FeSO_4_.7H_2_O, 0.02 g; trace element solution 1 ml; NaNO_3_, 0.45 mg; L-Cysteine HCl, 50 mg supplemented with 1% of dextrose or 0.05% of peptone or yeast extract. The dextrose solution was sterilized separately and added to the minimal medium after autoclave under aseptic conditions. All above media were prepared anaerobically (by purging with oxygen free nitrogen while boiling the medium) in serum vials and sealed under anaerobic conditions. Inoculation and sampling was done by using sterile syringes.

### Bacteriocin activity assay

For antimicrobial peptide production strain IE-3 was grown in anaerobic broth for 48 h under anaerobic conditions using nitrogen gas. The culture was incubated at 30°C with shaking at 120 rpm for optimal growth. The CFB obtained by removing the cells present in the medium by centrifugation (6,000 g for 10 min, 4°C) and subsequent filtration of the supernatant through 0.22 μm filter (Millipore, USA). The CFB was used to test the growth inhibition activity by agar well diffusion assay using actively growing test strains (between 0.2-0.4 OD). A growth curve verses antimicrobial production graph up to 48 h was constructed for strain IE-3 to examine the bacteriocin production at regular time intervals using anaerobic broth. Bacterial growth was measured as absorbance at 600 nm after constant time intervals of 2 h and antimicrobial activity at same time point was estimated by zone inhibition assay against *L. monocytogenes* test strain.

### Purification of low molecular weight antimicrobial peptide

Strain IE-3 was grown anaerobically in serum vials at 30°C for 48 h for the maximum production of a LMW peptide. Antimicrobial compound was extracted from CFB using 2% activated Diaion HP20 (Sigma, USA) hydrophobic resin. The crude extract obtained was further purified through cation exchange (Capto S, GE Healthcare, USA) chromatography column linked to an AKTA prime plus (GE healthcare, USA), in 20 mM sodium acetate buffer (pH 4.6) and eluted with NaCl gradient (50 to 1000 mM) in binding buffer. The peptide was desalted using dialysis tube (molecular cutoff 0.5 kDa, Spectrum, USA). Approximate molecular mass of peptide was determined by gel filtration column (Sodex KW-802.5) using standard molecular weight markers as described earlier [31]. Purity was confirmed by reversed phase HPLC (10 mm × 250 mm × 150 Å) C-18 column (venusil, Agela Technologies) under isocratic flow (1.5 ml/min) of acetonitrile (20%) along with 0.1% TFA. Elution was monitored at 200–340 nm wavelength range on PDA detector and peaks were collected by fraction collector (1260 Infinity, Agilent technology, USA).

### In-gel activity assay

The partially purified antimicrobial peptide (50 μg/lane) was electrophoresed in duplicate on 18.0% tricine SDS-PAGE [32]. One set of the gel lane along with protein ladder (multi-color low range protein ladder, Thermo Spectra™) was stained with Coomassie brilliant blue to confirm the location of the antimicrobial peptide and the other lane of the gel was used to test antimicrobial activity as described earlier [33] by overlaying with 5 ml of log-phase culture of *L. monocytogenes* (10^6^ cells/ml) and was incubated at 30°C overnight.

### Intact mass analysis and *de novo* sequencing

To analyze the molecular mass of peptide, purified peptide was electrophoresed, eluted from tricine SDS-PAGE by 75% acetonitrile with 0.1% TFA and used only for mass analysis and sequencing. Eluted peptide was mixed with equal ratios (1:1) of α-cyano-4-hydroxycinnamic acid in 50% acetonitrile and 0.1% (v/v) TFA. Samples were air dried and analyzed on an AB Sciex 5800 MALDI-TOF-TOF™ mass spectrometer. MS/MS data was acquired at 1000 Hz in 1 kV MSMS mode with 2000 laser shots/spectrum in CID (collision induced dissociation) mode to obtain maximum resolution. Sequence was generated by *de novo* explorer of AB Sciex and the highest score value sequence was considered as putative sequence. Further, structure was predicted on PEP-FOLD [34] server using *de novo* sequence. The structure obtained was visualized in PyMOL [35].

### Determination of minimum inhibitory concentration (MIC)

The MIC was determined for various indicator strains using a microtiter plate dilution assay as described earlier [31]. Cell growth was measured by observing OD at 600 nm at 16 h time interval using microtiter plate reader (Multiskan spectrum, Thermo, USA). The protein concentration was determined by BCA protein concentration estimation kit (Thermo, USA) following instructions of the manufacturer. For MIC determination of DTT treated peptide, the DTT solution was filter sterilized and final 100 mM concentration was used to treat peptide.

### Effect of pH, temperature, proteolytic enzymes, DTT and H_2_O_2_ on bacteriocin activity

The sensitivity of the bacteriocin towards different pH, temperatures and proteases was evaluated using purified bacteriocin. The purified peptide was incubated between pH values 2.0-10.0 and temperatures including 80, 100°C for 30 min and 120°C for 15 min. Antimicrobial peptide (200 μg) was incubated with various proteolytic enzymes such as trypsin (10 μg/ml, Sigma, USA), chymotrypsin (5 μg/ml, Sigma, USA) and proteinase K (5 units, Sigma, USA) in 100 mM Tris HCl buffer pH 8.0 (with 10 mM CaCl_2_) at 30°C for 6 h to determine their effect. The enzyme activity was terminated by heating the reaction mix at 80°C and subsequently used for antimicrobial activity assay. To test the effect of denaturant like DTT (BioRad, USA) on antimicrobial activity of the peptide, it was incubated with 50 to 150 mM DTT at room temperature for 1 h and used for growth inhibition assay. Hydrogen peroxide induced oxidation was tested by incubating the purified peptide with 100 mM concentration of hydrogen peroxide (Merck, India) for 1 h at room temperature [36] and activity was tested by well diffusion assay.

### Hemolysis assay

Blood was collected from New Zealand white rabbit, housed under normal conditions and had free access to a standard diet and water in Animal facility of the Institute. All animal protocols were followed according to the National Regulatory Guidelines issued by Committee for the Purpose of Control and Supervision of Experiments on Animals (CPCSEA), Ministry of Environment & Forests (Government of India). Red blood cells (RBCs) were separated from the whole blood by centrifugation (900 g) and washed twice with phosphate buffer saline (PBS). Washed cells resuspended into PBS and were counted using heamocytometer. For heamolysis, 2×10^8^ cells/ml were used as mentioned [37]. They were treated with different concentrations of purified peptide (ranging from 5 to 100 μM) and incubated in CO_2_ incubator for 24 h at 37°C. After incubation, cell free supernatant was separated by centrifugation (900 g) and absorbance was taken at 541 nm. PBS and triton X100 (0.1% v/v) were used as baseline and 100% lysis controls, respectively.

### Statistical analysis

Statistical significance of experimental results was determined by Student’s *t* test analysis and values of p < 0.05 were considered statistically significant. Data obtained from two individual experiments performed in triplicates was used.
